# Implementation of an Accelerated Infusion Protocol (90-Minute Infusion) of Rituximab and Its Safety in Patients With Autoimmune Rheumatic Diseases at a Tertiary Veterans Affairs Center

**DOI:** 10.7759/cureus.60558

**Published:** 2024-05-18

**Authors:** Vicky Nahra, David Panning, Donald Anthony, Maya Mattar

**Affiliations:** 1 Rheumatology, University Hospitals Cleveland Medical Center, Cleveland, USA; 2 Pharmacy, Veterans Affairs Medical Center, Cleveland, USA; 3 Immunology, Case Western Reserve University, Cleveland, USA; 4 Rheumatology, Veterans Affairs Medical Center, Cleveland, USA

**Keywords:** clinical rheumatology, accelerated protocol, reaction, infusion, rituximab

## Abstract

Background

Rituximab, a chimeric monoclonal antibody targeting the CD20 protein on the surface of B cells, is used to treat several rheumatologic and oncologic diseases. The standard infusion duration of rituximab is four hours.

Objective

Evaluating the safety of administering the accelerated 90-minute protocol at our Veterans Affairs center to patients with rheumatologic diseases and monitoring for any infusion-related reactions. This study is unique as it examines infusion rates faster than those most described (120 minutes).

Methods

Patients treated with rituximab for autoimmune diseases between June 2020 and June 2022 at our center were included in the study. Our patients were over 18 years of age, met the inclusion criteria, and had received previous rituximab infusions without prior infusion-related reactions. They received the accelerated protocol of 90 minutes over their next cycles and were monitored for any reactions during their infusions.

Results

A total of 34 patients receiving 76 infusions were included in the analysis. Most of the patients were males (n = 27). The most prevalent indication for rituximab infusion was rheumatoid arthritis (n = 20). Out of 76 infusions, only two infusion-related reactions were recorded (2.6% incidence). The first patient had itching and a sore throat, indicating a grade 1A reaction. The second patient developed chest pain and dyspnea, which resolved with diphenhydramine and albuterol. For both, the infusion was completed after appropriate management.

Conclusion

The incidence of infusion-related reactions during the accelerated 90-minute rituximab infusion was remarkably low and well-tolerated by our rituximab-experienced patients. Only two infusions were complicated by a reaction, an incidence comparable to or even lower than other reported 120-minute infusion protocols. This protocol is time- and cost-efficient, allowing for more infusions per chair per day at our center.

## Introduction

Rituximab (RTX) is a chimeric mouse/human immunoglobulin G1 (IgG1) monoclonal antibody that targets the transmembrane protein CD20. The latter is a protein expressed on the surface of most B cells that is thought to act primarily by depleting CD20-positive B cells [1]. This binding leads to apoptosis of these cells with antibody- and complement-dependent cytotoxicity. This mechanism of action leads to profound B-cell lymphopenia that persists for months following a single course of treatment. RTX is used for the treatment of various autoimmune and malignant diseases. It is FDA-approved for the treatment of non-Hodgkin’s lymphoma, chronic lymphocytic leukemia, rheumatoid arthritis (RA), microscopic polyangiitis, granulomatosis with polyangiitis, and pemphigus vulgaris. However, its safety and efficacy over the past 25 years have extended its use to other autoimmune diseases, such as IgG4-related disease, extra-glandular Sjogren’s syndrome, scleroderma, lupus nephritis, neuromyelitis optica, and anti-synthetase syndrome with interstitial lung disease.

In rheumatology clinical practice for the treatment of RA, RTX is administered at two doses of 1000 mg given two weeks apart [2,3]. For the treatment of other diseases like granulomatosis with polyangiitis or microscopic polyangiitis, different doses are used [2,4]. However, different regimens are used for maintenance depending on the experts’ recommendations, clinician preferences, and disease severity at presentation. The recommended approach for initial administration of the medication involves a slow infusion, with an initial rate of 50 mg/hour for 0.5 hours, followed by a gradual increase in the rate by 50 mg/hour every 0.5 hours, if it is well tolerated by the patient. The rate may ultimately be increased to a maximum of 400 mg/hour. Approximately 80% of fatal infusion-related reactions (IRRs) occur during the first infusion. Therefore, if the initial infusion is well-tolerated, subsequent infusions may be initiated at a rate of 100 mg/hour. The rate can then be increased by 100 mg/hour every 0.5 hours to a maximum of 400 mg/hour if there is no evidence of hypersensitivity [2]. IRRs to RTX occur at an incidence rate of up to 77% with the first infusion and are markedly less common after the initial infusion, as reported by the manufacturer [5-8]. Over time, several schemes have been proposed to classify hypersensitivity reactions associated with monoclonal antibody treatments [9-12]. The most described ones and their incidences include the following [12,13].

Type I reactions (immunoglobulin E (IgE)/non-IgE) are common reactions, with an incidence of approximately 63% and a clinical presentation that includes flushing, pruritus, urticaria, shortness of breath, wheezing, hypotension, and life-threatening anaphylaxis. The underlying mechanism is IgE- or non-IgE-mediated mast cell/basophil degranulation, which leads to the release of histamine, leukotrienes, and prostaglandins, causing allergic reactions.

Cytokine release reactions are less common with an incidence of 13% and a clinical presentation that includes fever/chills, nausea, pain, headache, and rigors not responding to premedication/slower infusion rate during the first infusion. The underlying mechanism is cytokine release with elevated serum tumor necrosis factor-alpha (TNF-α) and interleukin 6 (IL-6) concentrations.

IRRs are common acute infusion reactions that occur shortly after infusion, with a prevalence of 20-50%. Their clinical presentation is like that of type 1 or cytokine release. They are usually mild and subside with the following infusions. Their pathogenesis is not clear, with a possible role for IL-6 and TNF-α likely due to the rate of infusion.

Mixed reactions are a combination of cytokine release and IgE-mediated reactions, with a prevalence of 21%. Their clinical presentation includes wheezing, flushing, urticaria, pruritus, fever/chills, nausea, pain, headache, and rigor.

Serum sickness reactions or type III reactions are less common, mainly seen with underlying rheumatic diseases like Sjogren’s disease. The classic triad of fever, rash, and arthralgia was reported to be seen in up to 48.5% of cases with serum sickness. The mechanism involves complement-fixing IgM and IgG antibodies targeted at the immunogenic part of RTX. The symptoms are usually benign and self-limiting [14].

Type IV or delayed reactions, with an incidence of 3%, usually present as a maculopapular rash [15]. Severe cutaneous reactions may occur but are rare, including Stevens-Johnson syndrome (SJS) and toxic epidermal necrolysis (TEN) [16].

Multiple grading systems exist for assessing the severity of hypersensitivity reactions to RTX or other monoclonal antibody treatments, including Brown [17] and the National Cancer Institute Grading System. The standard rate of RTX infusion is both labor- and time-intensive, with an average infusion time of approximately four to five hours for the first infusion and three hours for subsequent infusions [18]. Medications are regularly used prior to infusion to reduce the frequency and severity of infusion reactions during infusions. Intravenous methylprednisolone 100 mg is indicated with the first infusion and may be administered with subsequent infusions. Acetaminophen and antihistamines may also be administered, although there is no rigorous evidence for the efficacy of antihistamines [19]. Multiple studies have established the safety of faster RTX protocols (60-90 minutes and 120 minutes) in both oncology and rheumatology practice settings [18,20-22]. The main study for non-oncology patients, RATE-RA, infused RTX as fast as 120 minutes. However, oncology studies have shown that faster rates (60-90 minutes) are safe, and an accelerated 90-minute rate was added to the package insert for RTX, but only for two indications: follicular lymphoma and diffuse large B-cell lymphoma. Moreover, these studies have shown fewer IRRs with faster infusion rates without serious events than the rates described with longer infusion protocols.

As a quality improvement initiative for our tertiary Veterans Affairs (VA) Medical Center infusion clinic patients, we evaluated the utilization of RTX at a 90-minute accelerated infusion rate. To reduce the chair time for our infusion patients, we developed and implemented a pharmacist-driven protocol that permits pharmacists to switch eligible patients from the regular infusion protocol to the 90-minute accelerated protocol. This study aimed to describe the incidence, types, and severity of IRRs related to the accelerated infusion protocol and assess its safety for future use in rheumatology practice settings.

## Materials and methods

This is a retrospective chart review study. All patients treated with RTX for RA and other autoimmune diseases, including off-label use, between June 2020 and June 2022, who met our eligibility criteria were selected to receive the RTX 90-minute accelerated infusion protocol. A total of 34 patients (76 infusions) were included in the study.

Patients who met the following eligibility criteria were included in the study: aged 18 years and above; rheumatology indication (RA and other auto-inflammatory/auto-immune conditions); must have had the previous dose of RTX within the last six months; dose must be 375 mg/m2 or less, or 1000 mg or less if using a fixed dose; absolute lymphocyte count less than 5000 cells per cubic meter; no grade 3 or higher infusion-related reaction with previous doses; no New York Heart Association (NYHA) heart failure class 3 or higher; no history of significant arrhythmia; no serious uncontrolled hypertension.

The study was approved by the Institutional Review Board (IRB) of our VA Medical Center and appropriate guidelines were followed. Informed consent from the patients was deemed exempt.

Infusion protocol

After meeting our inclusion criteria, patients received their next scheduled RTX infusion as accelerated. RTX was administered at the rate specified in Table 1, with 20% of the dose given over the first 0.5 hours and the remaining 80% infused over one hour. Premedication agents included diphenhydramine and acetaminophen. Methylprednisolone was optionally administered for the patient’s first accelerated dose (Table 1).

**Table 1:** Rituximab accelerated infusion rate, study protocol. mg = milligrams; ml = milliliters.

90-minute accelerated rituximab rate		
Dose	Initial, 30-min rate	Last, 60-min rate
500 mg, 125 mL	50 mL/hour	100 mL/hour
600 mg, 150 mL	60 mL/hour	120 mL/hour
700 mg, 175 mL	70 mL/hour	140 mL/hour
800 mg, 200 mL	80 mL/hour	160 mL/hour
900 mg, 225 mL	90 mL/hour	180 mL/hour
1000 mg, 250 mL	100 mL/hour	200 mL/hour
1100 mg, 275 mL	110 mL/hour	220 mL/hour

Infusion-related reactions

We monitored the patients for adverse events or reactions during their first accelerated and subsequent infusions. The modified oncologic version of the National Cancer Institute Common Terminology Criteria for Adverse Events Scale was used to describe the severity of reactions, with scores ranging from 1 (mild reaction) to 4 (severe reaction) [23]. Two different physicians were present at the infusion clinic during medication administration and assigned the grading. According to the modified grading scale, the grades were defined as follows: grade 1A: cutaneous symptoms only (rash, itching, or flushing); grade 1B: cutaneous symptoms in addition to back pain and/or hypertension; grade 2: urticaria, nausea, vomiting, throat tightness, asymptomatic bronchospasm, and/or chest tightness; grade 3: symptomatic bronchospasm, dyspnea, hypoxia, and/or wheezing; grade 4: anaphylaxis or hypotension; grade 5: death.

## Results

Our study included 34 patients, most of whom received more than one accelerated infusion during the study period. Most of the patients were males, reflecting our larger VA population (Table 2). The most prevalent indication for RTX infusion at our center was RA, which is similar to the findings in other studies [24,25]. A total of 76 infusions were administered from June 2020 to June 2022, and of these infusions, only two IRRs were recorded (Table 3), which is equal to an incidence of 2.6% (two IRRs out of 76 infusions). The first patient who experienced an IRR had itching and sore throat around 25 minutes into the infusion, which was classified as a grade 1A reaction according to the grading system. The patient received diphenhydramine and acetaminophen as premedication without methylprednisolone. The infusion was interrupted, and 100 mg of hydrocortisone was administered as part of the hypersensitivity protocol. The patient’s symptoms improved, and the infusion was resumed at a lower rate and completed. Subsequent infusions were tolerated at an accelerated rate, with premedication administered before the infusions, including methylprednisolone, acetaminophen, and antihistamines, and no subsequent reactions. The patient’s indication for RTX treatment was lupus nephritis.

**Table 2:** Patient demographics and clinical characteristics. SD = standard deviation; ANCA = antineutrophilic cytoplasmic antibody; IgG4 = immunoglobulin G4; ILD = interstitial lung disease.

Autoimmune disease	Rheumatoid arthritis	Sjogren’s syndrome	ANCA vasculitis	IgG4-related disease	Lupus nephritis	Scleroderma	Neuromyelitis optica	Anti-synthetase syndrome with ILD
Number of patients	20	2	4	2	2	2	1	1
Female	2	2	1	0	2	0	0	0
Male	18	0	3	2	0	2	1	1
Age (mean ± SD, in years)	66.7 ± 10.1	66 ± 5.65	61.25 ± 13.07	56 ± 12.7	48 ± 11.3	64.5 ± 9.2	28	73
Disease duration (mean ± SD, in years)	14.7 ± 9.88	13.5 ± 10.6	2.75 ± 2.87	3.5 ± 2.12	17.5 ± 0.7	6 ± 0	7	4

**Table 3:** Total infusions of rituximab per rheumatologic indication, including the number of patients per indication, receipt of methylprednisolone, and the presence of an infusion reaction. ANCA = antineutrophilic cytoplasmic antibody; IgG4 = immunoglobulin G4; ILD = interstitial lung disease.

Indication	Unique patients	Total infusions	Methylprednisolone receipt during infusion?				Infusion reaction	
			No	Yes				
				20 mg	40 mg	62.5 mg	No	Yes
ANCA-associated vasculitis	4	8	8	0	0	0	8	
Anti-synthetase syndrome with ILD	1	2	2	0	0	0	2	
IgG4-related disease	2	3	3	0	0	0	3	
Lupus nephritis	2	2	1	0	1	0	1	1
Neuromyelitis optica	1	2	2	0	0	0	2	
Rheumatoid arthritis	20	51	43	4	2	2	50	1
Scleroderma	2	3	2	0	1	0	3	
Sjogren's syndrome	2	5	5	0	0	0	5	
Total	34	76	66	4	4	2	74	2

The second patient who experienced IRR developed chest pain and dyspnea approximately one hour into the infusion, which is classified as a grade 3 reaction. The patient had received diphenhydramine and acetaminophen as premedication without methylprednisolone. The infusion was interrupted, and the patient was administered oxygen through a nasal cannula, albuterol, diphenhydramine 25 mg twice, and intravenous fluid. After a 30-minute pause, the patient’s condition improved, and the infusion was resumed at a lower rate and was tolerated until it was completed. The subsequent infusion was administered at a slower rate due to the grade 3 reaction and the patient’s history of systolic heart failure with NYHA class 1-2, in addition to acetaminophen and diphenhydramine, but complicated by atrial fibrillation, and infusions were stopped permanently. Earlier on, this patient had previously tolerated one infusion at a faster rate of RTX for the treatment of RA without any IRRs but subsequently had an IRR as mentioned. The incidence of IRR in our study was 2.6%, and two out of the 76 infusions had an IRR. The other 74 infusions were free of IRRs.

## Discussion

Thirty-four patients with autoimmune disorders who received a total of 76 infusions of the accelerated 90-minute protocol of RTX were included in the study. It was over a two-year period. The patients had previous treatment experience with RTX without adverse events. The calculated IRR incidence in this study was 2.6% per infusion. Our reaction rates are comparable to those of other studies, and significantly lower than most of them that also utilized RTX in an accelerated dose protocol and monitored for safety signals. The RATE-RA study by Pritchard et al. evaluated the incidence of IRR in 351 RA patients on an accelerated RTX protocol infused over 120 minutes, for a duration of 30 weeks. The results showed an IRR incidence ranging from 0.7% to 16.2%, depending on the number of infusions received, from one to four infusions [18]. Fenton et al. performed a study similar to ours at a VA medical center, with patients (malignant and non-malignant indications) receiving the accelerated 90-minute RTX protocol. Eleven patients received 24 infusions over a five-month period. The incidence of IRR is 4.2% per infusion [20]. Another study by Can et al. examined 68 patients with rheumatologic diseases receiving the accelerated 120-minute RTX protocol, including RA, systemic lupus erythematosus, and vasculitis, totaling up to 77 infusions. They reported an IRR incidence of 14.7% per infusion [24]. Larsen et al. investigated the accelerated 90-minute RTX protocol in 54 patients with diverse autoimmune diseases, a total of 108 infusions, over a period of two months and reported an incidence of 18.5% per patient [25]. In a study by Hartinger et al., 53 patients with autoimmune diseases with renal involvement and selected primary glomerulonephritis received a 120-minute RTX protocol. A total of 85 infusions were administered, with only two reported IRRs, with an incidence of 2.35% per infusion [26]. Another study by Patel et al. evaluated the IRR in a population of 109 patients with hematologic malignancies (low-grade lymphoma), receiving a total of 647 accelerated 90-minute protocol RTX infusions over a period of four years. They reported four infusion-related reactions with an incidence of 0.61% per infusion [27]. All IRRs experienced at our center were mild to moderate, with no severe reactions. The infusions were resumed and completed after appropriate pharmacological treatment, resulting in no interruption of RTX treatment. Our protocol successfully reduced the time spent in the chair from approximately four hours to 90 minutes, without significant adverse events.

## Conclusions

The main purpose of our study was to safely improve the time spent during RTX infusions for both our patients and our center to increase the center’s capacity to accommodate and treat other patients with rheumatologic diseases. Our results showed that the incidence of IRR during the accelerated 90-minute RTX infusion was significantly low and well-tolerated by our RTX treatment-experienced patients, with only two infusions complicated by an IRR, with an incidence of 2.6% per infusion. The IRRs were mild (lower than grade 4), and there was no need for hospitalization. The patients with IRR were still able to complete their infusions after appropriate treatment. Our study has multiple unique features. We used the 90-minute RTX infusion protocol rather than the 120-minute protocol, so it was faster. Also, it was done over a duration of two years, longer than other similar studies. In addition, our population included only patients with autoimmune diseases, in contrast to hematology/oncology patients or mixed populations.

A limitation of our study is the small number of patients, as well as the fact that our patients had long-standing experience with RTX treatment; therefore, caution should be taken when extrapolating to patients more recently started on RTX. Future efforts to evaluate advancing the 2nd or 3rd treatment cycle to the accelerated infusion protocol would be valuable in patients who are actually RTX-naïve. Also, this study only included adult patients, with no current data on patients under 18 years of age. We also lack data on the incidence of infusion-related reactions for patients on the standard infusion rate of rituximab that is prior to the start of the accelerated protocol. The accelerated protocol is safe in RTX-experienced patients, time- and cost-efficient, and beneficial for infusion centers by increasing both the number of infusion chairs available per day and nursing efficiency. In addition, it is more convenient and time-saving for patients.
